# Case Report: Sigmoidorectal intussusception mimicking rectal prolapse—a diagnostic pitfall with surgical implications

**DOI:** 10.3389/fsurg.2026.1852407

**Published:** 2026-07-08

**Authors:** Tudor-Alexandru Popoiu, Dan Brebu, Rares Borcean, Adrian Vaduva, Mircea Selaru

**Affiliations:** 1 Department of Functional Sciences, Medical Informatics and Biostatistics Discipline, “Victor Babes” University of Medicine and Pharmacy Timisoara, Timisoara, Romania; 2 Doctoral School, Faculty of Medicine, “Victor Babes” University of Medicine and Pharmacy Timisoara, Timisoara, Romania; 3 IIIrd Surgery Clinic, “Pius Brinzeu” County Emergency Clinical Hospital Timisoara, Timișoara, Romania; 4 IInd Surgery Clinic, Timișoara Emergency County Hospital, Timisoara, Romania; 5 X Department of General Surgery, “Victor Babeș” University of Medicine and Pharmacy, Timisoara, Romania; 6 Discipline of Surgical Emergencies, Department of Surgery II, Victor Babes University of Medicine and Pharmacy, Timisoara, Romania; 7 Department of Microscopic Morphology-Morphopathology, ANAPATMOL Research Center, “Victor Babeş” University of Medicine and Pharmacy, Timișoara, Romania; 8 Department of Pathology, “Pius Brînzeu” County Clinical Emergency Hospital, Timişoara, Romania

**Keywords:** adult intussusception, colorectal surgery, computed tomography, lead point lesion, rectal prolapse mimicry, sigmoidorectal intussusception

## Abstract

Adult intussusception is a rare condition typically associated with a structural lead point and often requiring surgical management, particularly in colonic forms where malignancy risk is high. Sigmoidorectal intussusception represents an exceptionally uncommon subtype that may clinically mimic rectal prolapse, posing a significant diagnostic challenge. We report the case of a 57-year-old female presenting with an irreducible anorectal mass, initially suggestive of prolapse, in whom computed tomography suggested the diagnosis of sigmoidorectal intussusception, which was subsequently confirmed intraoperatively. The patient underwent emergency surgical resection with primary anastomosis, and histopathology revealed a large tubulovillous adenoma with high-grade dysplasia as the lead point. The postoperative course was uneventful. This case highlights the importance of maintaining a high index of suspicion for intussusception in atypical prolapsing anorectal lesions and underscores the critical role of cross-sectional imaging in differentiating between rectal prolapse and invagination. Timely diagnosis enables appropriate oncologic surgical management and helps avoid potentially inappropriate perineal approaches.

## 1 Introduction

Adult intussusception is a rare and diagnostically challenging condition, accounting for approximately 5% of all intussusception cases and 1% of bowel obstructions (1, 2). Unlike pediatric forms, it is typically associated with an identifiable structural lead point and frequently requires surgical treatment. Colonic intussusception carries a particularly high risk of malignancy, making accurate diagnosis essential (3, 4).

Sigmoidorectal intussusception represents an exceptionally uncommon subtype that may present with a prolapsing anorectal mass, closely mimicking primary rectal prolapse (5). This overlap may lead to misdiagnosis and inappropriate management, particularly if surgical decisions are based solely on clinical examination.

We report a case of sigmoidorectal intussusception caused by a large adenomatous polyp presenting as an irreducible anorectal mass, highlighting key diagnostic pitfalls and the role of computed tomography in guiding management.

## 2 Case presentation

A 57-year-old female with no significant past medical history presented to the colorectal surgery service with acute exteriorization of a tumoral mass through the anal canal, accompanied by local pain and minor rectal bleeding. The symptoms had developed abruptly 48 h prior to presentation and progressively worsened, prompting emergency evaluation.

The patient reported a screening colonoscopy performed two years earlier, which revealed no pathological findings except for a sigmoid polyp measuring approximately 1 cm. The lesion was endoscopically resected, and histopathological examination at that time demonstrated a tubulovillous adenoma, without evidence of invasive malignancy. No interval surveillance colonoscopy was documented following the initial polypectomy, and the relationship between the previously resected lesion and the current tumor could not be definitively established.

### 2.1 Clinical examination

**Figure 1:**
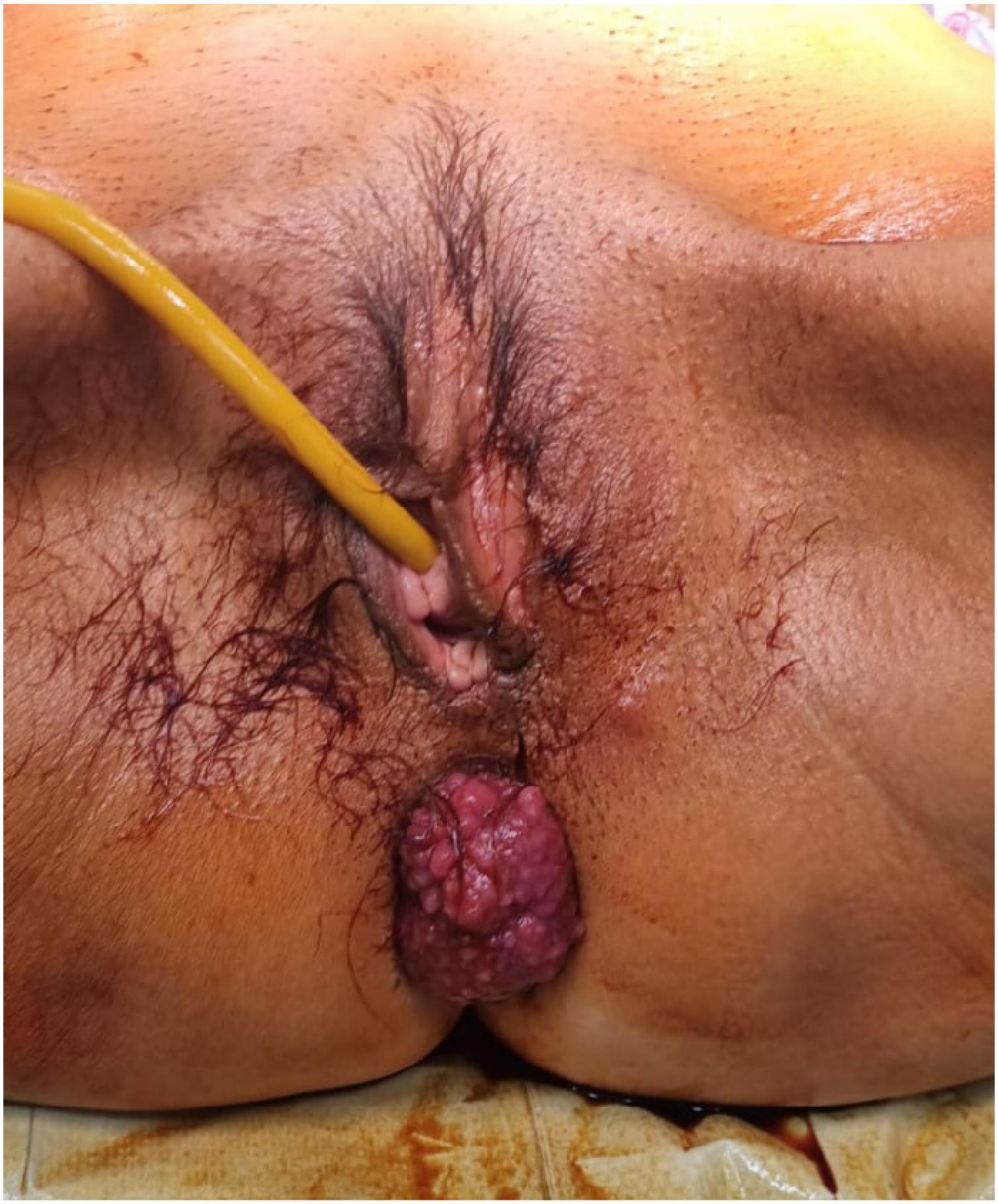
Clinical examination of the perianal region.

On physical inspection, a large, edematous, friable, cauliflower-shaped mass was observed protruding through the anal canal, measuring approximately 6 cm in diameter (Figure 1). The lesion was tender on palpation and exhibited areas of superficial bleeding. Digital rectal examination did not reveal additional intraluminal lesions within the anal canal; however, manual reduction of the prolapsed mass was not possible, raising suspicion for a pathology other than primary rectal prolapse.

### 2.2 Laboratory investigations

Initial laboratory evaluation showed a leukocyte count of 9.68 × 10³/µL (reference range: 4–10 × 10³/µL) with neutrophil predominance (72.7%). Hemoglobin and hematocrit values were within normal limits, excluding significant blood loss. A reactive thrombocytosis (420 × 10³/µL; reference range: 150–400 × 10³/µL) was noted. Inflammatory markers were not significantly elevated, and C-reactive protein remained within the normal range. Serum electrolytes revealed mild hyponatremia, while renal and hepatic function tests were within normal limits.

Overall, the laboratory findings were nonspecific and did not indicate systemic inflammation or advanced disease. The absence of bowel ischemia was supported by the clinical presentation, imaging findings, and intraoperative assessment.

### 2.3 Imaging studies

**Figure 2:**
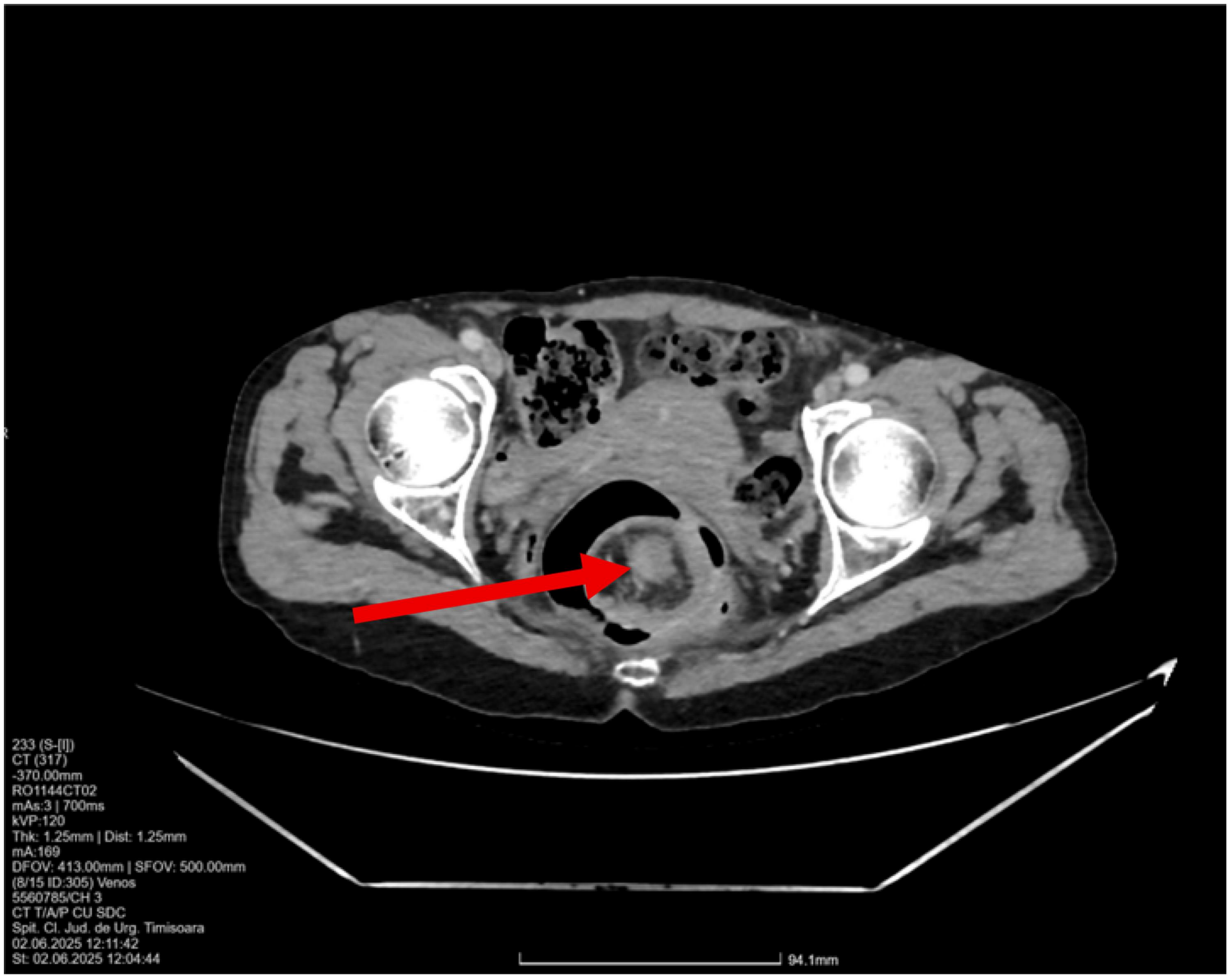
Axial contrast-enhanced CT slice through the pelvis demonstrates a bowel-within-bowel configuration within the rectal lumen, with concentric rings (“target” or “donut” sign) and invaginated mesenteric fat. The surrounding pelvic bones (iliac wings and acetabula) are visible.

**Figure 3:**
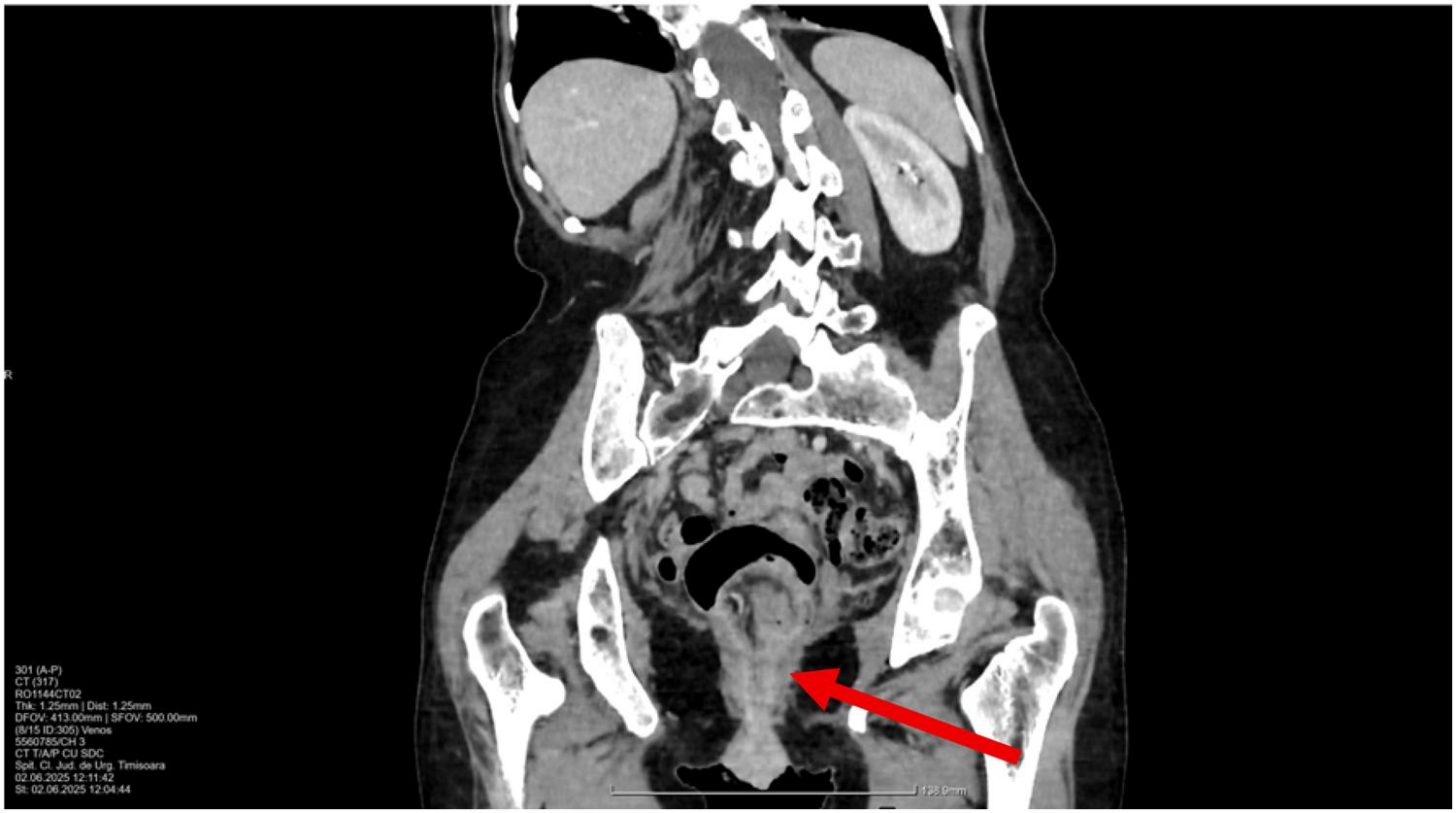
Coronal reconstruction shows a segment of telescoped bowel extending into the rectum, again demonstrating the characteristic layered appearance of intussusception. The invaginated segment appears to extend from the sigmoid colon into the rectum. No obvious free intraperitoneal air is visible on this slice.

A contrast-enhanced computed tomography (CT) scan of the thorax, abdomen, and pelvis was performed for diagnostic evaluation. Imaging revealed a heterogeneous, irregular, contrast-enhancing, cauliflower-like mass protruding through the anal canal, measuring approximately 44 × 39.5 mm. The findings were interpreted as suggestive of an incomplete sigmoidorectal intussusception or prolapse, without radiological signs of complete bowel obstruction, perforation, or ischemia. No abdominal or pelvic lymphadenopathy was identified. The liver and spleen demonstrated multiple hypodense lesions with benign imaging characteristics, considered incidental findings without clinical relevance to the present condition, and therefore not contributory to the diagnostic or therapeutic approach. No distant metastatic disease was detected (Figures 2, 3).

Given the atypical anorectal presentation, irreducibility of the prolapsed segment, and imaging findings suggestive of invagination rather than primary prolapse, surgical intervention was indicated.

### 2.4 Surgical treatment

Given the atypical presentation with an irreducible prolapsing anorectal mass and imaging findings suggestive of intussusception, emergency surgical intervention was indicated. An open approach via midline laparotomy was chosen due to the acute clinical presentation, the need for rapid and comprehensive intra-abdominal assessment, and the limited institutional experience with laparoscopic management of such rare and complex cases.

Intraoperatively, a sigmoidorectal intussusception was confirmed, with the sigmoid colon telescoping into the rectum. Careful manual reduction was performed, revealing a pedunculated intraluminal lesion acting as the lead point. A segmental sigmoid resection was carried out, including the involved bowel segment and its associated mesentery. Reconstruction was performed using a mechanical termino-lateral colorectal anastomosis with a circular stapling device. This configuration was selected to facilitate a tension-free anastomosis with adequate vascular perfusion and to better accommodate differences in luminal diameter between the proximal colon and distal rectum following reduction. Additionally, the termino-lateral approach allows for favorable alignment of the bowel segments and may reduce the risk of anastomotic tension in this anatomical setting. Anastomotic integrity was confirmed intraoperatively.

### 2.5 Histopathological findings

**Figure 4:**
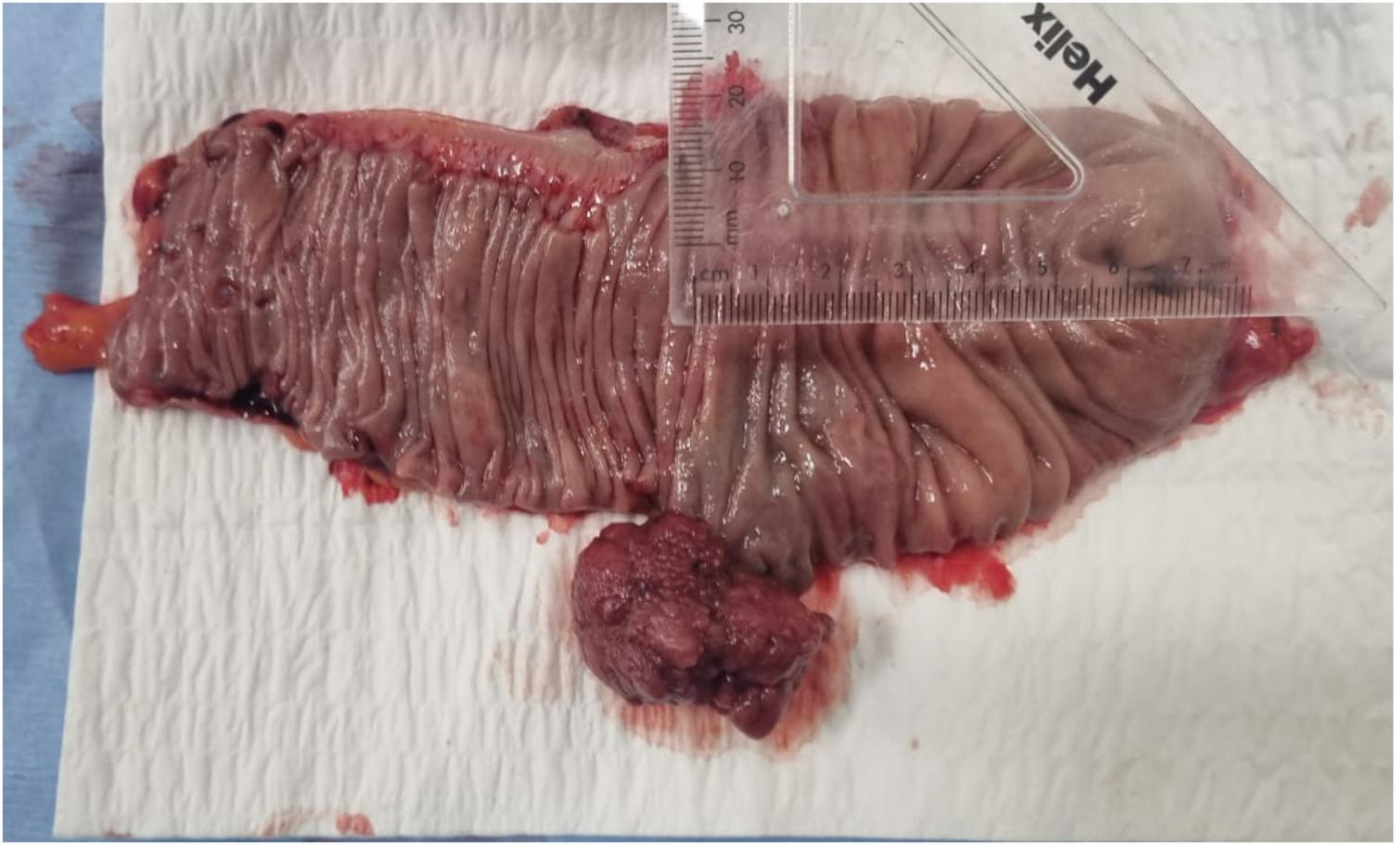
Resected sigmoid colon specimen showing the pedunculated polyp serving as the lead point of the intussusception.

**Figure 5:**
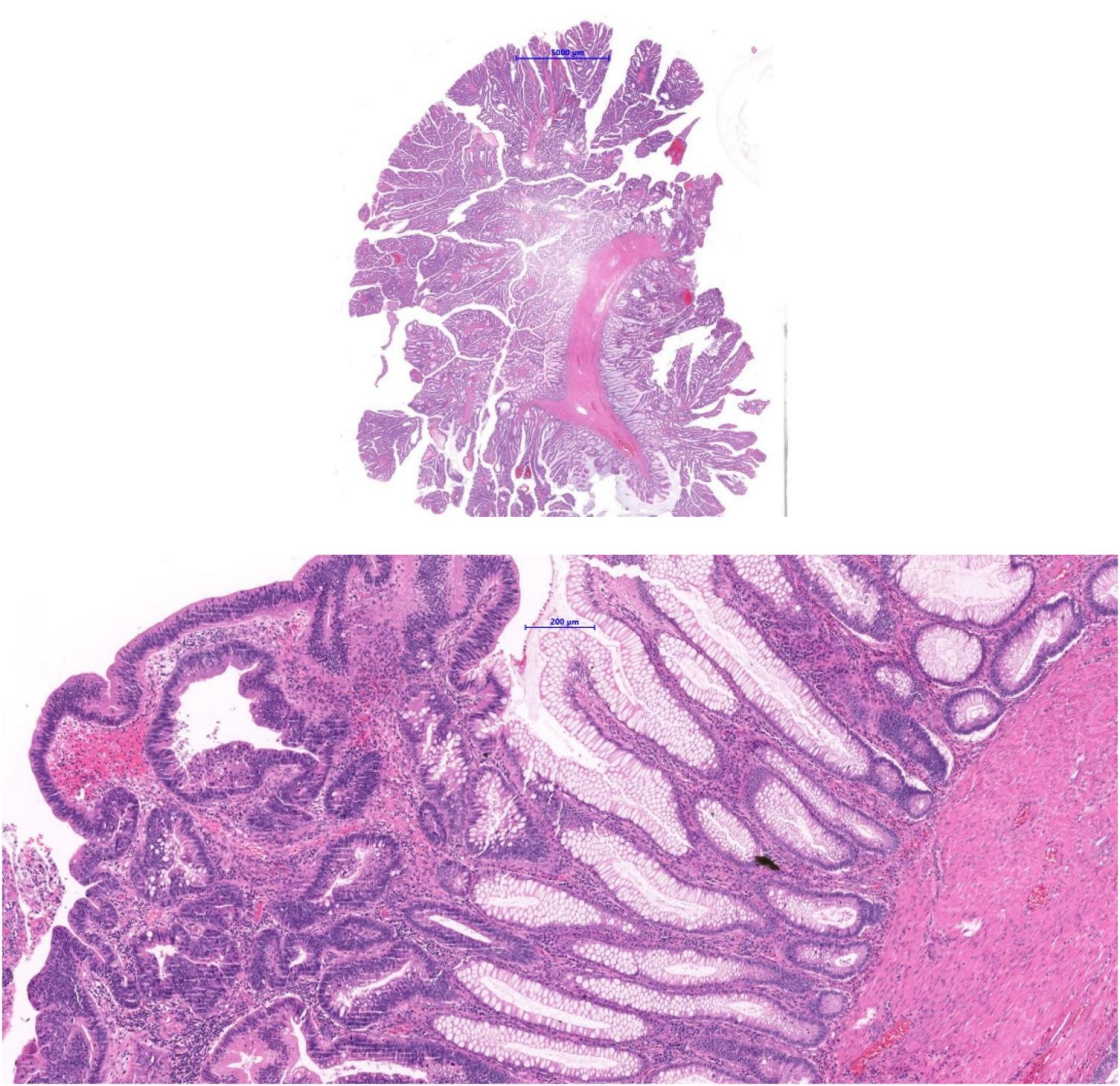
Low magnification showing a tubulovillous polyp with dysplastic areas on the surface; high power view showing dysplastic changes on the surface without invasion.

Gross examination of the resected specimen demonstrated a pedunculated polypoid lesion measuring 4.0 × 3.8 × 1.2 cm, located 7 cm from the proximal and 4.5 cm from the distal resection margins (see Figure 4). Microscopic analysis revealed a tubulovillous adenomatous polyp with extensive low-grade dysplasia and focal areas of high-grade dysplasia (Figures 5A,B), without evidence of invasive carcinoma. Thirteen pericolic lymph nodes were identified and were free of pathological involvement, and all surgical margins were uninvolved.

### 2.6 Postoperative course and follow-up

The postoperative course was uneventful, with prompt restoration of bowel function and no surgical or medical complications. The patient was discharged in good general condition. At early follow-up, she remained asymptomatic, with no evidence of recurrent prolapse, bleeding, or obstructive symptoms.

### 2.7 Patient perspective

**Figure 6:**
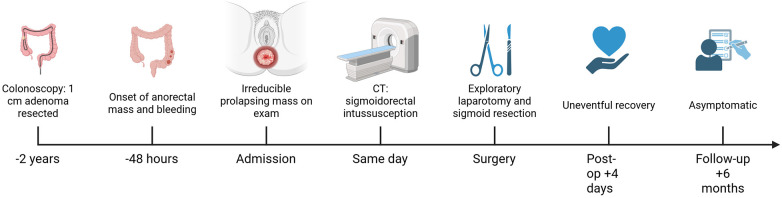
Clinical timeline of diagnosis and surgical management. Created in BioRender. Popoiu, T. (2026) https://BioRender.com/ljeq9ey.

The patient reported significant anxiety related to the acute presentation and the unexpected nature of the diagnosis. She expressed satisfaction with the surgical outcome and the resolution of symptoms following treatment. The short overview over the case timeline can be seen in Figure 6.

## 3 Discussion

Adult intussusception represents a rare and diagnostically challenging clinical entity, particularly when involving the distal colon and rectum. Sigmoidorectal intussusception is an exceptionally uncommon subtype and remains underrepresented in large case series, largely confined to isolated case reports. Its clinical relevance lies not only in its rarity but also in its potential to masquerade as more common anorectal conditions, most notably primary rectal prolapse, thereby increasing the risk of diagnostic delay and inappropriate management (6, 7).

The present case illustrates several features that underscore these challenges. First, the patient presented with acute exteriorization of a friable anorectal mass, a presentation that strongly suggests rectal prolapse in routine clinical practice. However, the acute onset, associated pain, bleeding, and irreducibility of the prolapsed segment were atypical for primary prolapse and should prompt consideration of alternative diagnoses. As highlighted in previous reports, sigmoidorectal intussusception frequently lacks the uniform concentric folds characteristic of true rectal prolapse and may instead present with an asymmetric, irregular mass with a discernible leading lesion—features observed in our patient (8).

Second, this case reinforces the pivotal role of computed tomography in establishing the correct diagnosis (9, 10). Despite advances in imaging, a substantial proportion of adult intussusceptions continue to be diagnosed intraoperatively. In the present case, CT imaging clearly demonstrated a colo-colic intussusception with the sigmoid colon invaginating into the rectum and prolapsing through the anal canal, with a pedunculated lesion identified as the lead point. This preoperative diagnosis was essential in guiding appropriate surgical planning and avoiding attempts at perineal reduction or prolapse directed procedures that would have been oncologically inappropriate.

Third, although malignant lesions account for most reported sigmoidorectal intussusceptions (8, 11), this case highlights that large benign lesions may produce an identical clinical and radiological picture. The identification of a sizeable adenomatous polyp as the lead point aligns with previous observations that lesion size, rather than histologic malignancy alone, is a critical determinant of telescoping and obstruction. Nevertheless, the strong association between colonic intussusception and malignancy justifies adherence to oncologic surgical principles irrespective of preoperative suspicion. The relationship between the previously resected adenoma and the current lesion remains uncertain. In the absence of interval endoscopic surveillance, it is not possible to determine whether this represents recurrence, incomplete prior resection, or the development of a new lesion. This limitation highlights the importance of appropriate post-polypectomy surveillance in patients with advanced adenomatous pathology.

From a therapeutic perspective, surgical resection remains the cornerstone of management in adult colonic and sigmoidorectal intussusception. Although intraoperative reduction was safely performed in this case to facilitate resection and reconstruction, definitive segmental resection with negative margins was essential to exclude malignancy and prevent recurrence. In recent years, minimally invasive approaches, particularly laparoscopic surgery, have been increasingly reported in the literature, demonstrating comparable safety and feasibility in selected patients (12–14). However, in the present case, an open approach was chosen due to the emergent clinical presentation and the limited institutional experience with laparoscopic management of such rare and complex conditions. The favorable postoperative course observed further supports timely surgical intervention once the diagnosis is established.

The main strength of this case lies in the rarity of sigmoidorectal intussusception presenting as an apparent rectal prolapse, a clinical scenario that carries a significant risk of misdiagnosis and inappropriate management. This unusual presentation underscores an important diagnostic pitfall in colorectal practice. Additionally, the clear correlation between clinical findings, cross-sectional imaging, and intraoperative confirmation strengthens the validity of the diagnosis and highlights the value of computed tomography in guiding appropriate management and avoiding potentially inappropriate perineal interventions. Limitations include the single-case nature of the report and the limited generalizability of findings, given the rarity of sigmoidorectal intussusception and the predominance of low-level evidence in the literature.

In conclusion, sigmoidorectal intussusception is a rare but important differential diagnosis in adults presenting with an irreducible anorectal mass. This case highlights the risk of misdiagnosis as primary rectal prolapse, particularly in the presence of atypical features such as acute onset, pain, bleeding, and irreducibility. Computed tomography plays a central role in suggesting the diagnosis and guiding appropriate management. Surgical resection remains the treatment of choice given the significant likelihood of an underlying pathological lead point. Early recognition and an imaging-guided approach are essential to avoid inappropriate interventions and ensure optimal outcomes.

## 4 Patient perspective

The patient reported significant anxiety related to the acute presentation and the unexpected nature of the diagnosis. She expressed satisfaction with the surgical outcome and the resolution of symptoms following treatment*.*

## Data Availability

The original contributions presented in the study are included in the article/Supplementary Material, further inquiries can be directed to the corresponding author.
